# Region-specific complexity of the intracranial EEG in the sleeping human brain

**DOI:** 10.1038/s41598-021-04213-8

**Published:** 2022-01-10

**Authors:** Elzbieta Olejarczyk, Jean Gotman, Birgit Frauscher

**Affiliations:** 1 Nalecz Institute of Biocybernetics and Biomedical Engineering, Polish Academy of Sciences, Trojdena 4 Str., 02-109 Warsaw, Poland; 2 Montreal Neurological Institute and Hospital, McGill University, Montreal, QC H3A 2B4 Canada

**Keywords:** Physiology, Medical research, Neuroscience, Circadian rhythms and sleep, Computational neuroscience

## Abstract

As the brain is a complex system with occurrence of self-similarity at different levels, a dedicated analysis of the complexity of brain signals is of interest to elucidate the functional role of various brain regions across the various stages of vigilance. We exploited intracranial electroencephalogram data from 38 cortical regions using the Higuchi fractal dimension (HFD) as measure to assess brain complexity, on a dataset of 1772 electrode locations. HFD values depended on sleep stage and topography. HFD increased with higher levels of vigilance, being highest during wakefulness in the frontal lobe. HFD did not change from wake to stage N2 in temporo-occipital regions. The transverse temporal gyrus was the only area in which the HFD did not differ between any two vigilance stages. Interestingly, HFD of wakefulness and stage R were different mainly in the precentral gyrus, possibly reflecting motor inhibition in stage R. The fusiform and parahippocampal gyri were the only areas showing no difference between wakefulness and N2. Stages R and N2 were similar only for the postcentral gyrus. Topographical analysis of brain complexity revealed that sleep stages are clearly differentiated in fronto-central brain regions, but that temporo-occipital regions sleep differently.

## Introduction

The study of brain organization and the relation between its anatomy and function has been of interest to neuroscientists since more than a century. In 1909, Korbinian Brodmann classified the brain cortical areas relying on neuron cytology^1^. The introduction of advanced brain imaging techniques such as Magnetic Resonance Imaging (MRI) allowed to develop even more detailed anatomical atlases of the brain^2–6^. The advantage that electrophysiological techniques, scalp electroencephalography (EEG) and magnetoencephalography (MEG), have over other neuroimaging techniques is that they enable signal recording with high temporal resolution (on the order of one millisecond) and that they reflect neuronal activity directly. The disadvantage of these methods is their low spatial resolution (one cm) and that they do not allow the examination of deep brain structures, as they measure the signal generated predominantly in the cerebral cortex located close to the brain surface^7^. Moreover, source reconstruction will always have some ambiguity because the inverse problem is ill-posed^8,9^. The use of intracranial EEG (iEEG) enables to overcome these pitfalls. It is, however, an invasive technique and in humans, prolonged iEEG recording is only performed in the context of pre-surgical epilepsy evaluation in patients with focal drug-resistant epilepsy. Since not all investigated brain areas demonstrate a pathological activity, it is possible to choose some iEEG channels for every patient showing an almost normal brain activity^10^. Multicenter collaboration is necessary to collect enough data from each of the various brain regions to obtain statistically significant results^11–14^.

The physiological basis of HFD is the brain’s complexity. Patterns of brain activity are self-similar at different spatial and temporal scales and at different levels of structural and functional organization, which is known as the fractal nature of the brain^15–18^. The fractal properties of a signal are studied using measures of complexity derived from nonlinear dynamics called also the theory of deterministic chaos. The Higuchi fractal dimension (HFD) was defined by Tatsuo Higuchi in 1988^19^. This method is particularly useful for EEG signal analysis. In 1994, HFD was applied for the first time to EEG in order to study the changes in the complexity of alpha waves from wakefulness to drowsiness by Inouye et al.^20^ HFD provides additional information to that provided by traditional spectral analysis that does not allow to capture non-linear phenomena characteristic for complex nervous systems and does not take into account the instability of the EEG signal. The HFD depends on the frequency composition of the signal. It gives higher values for higher frequencies and vice-versa. However, in contrast to traditional spectral analysis, it is a global measure which allows to capture phase relations in the EEG signals, and provides information about the relations between the components of different frequency bands. In comparison to other nonlinear measures requiring phase space reconstruction, computation of the HFD is a simple and fast. It is defined directly in the time domain and it can be calculated in short time intervals on the order of hundred samples independently of the sampling frequency. In the first decade of 2000s Klonowski and Olejarczyk, followed by their collaborators and other investigators, successfully disseminated the HFD method. They showed that this single measure of the complexity may be useful in the differentiation of various physiological and pathological states, including sleep^21–26^, anesthesia^25,27–30^, epilepsy^25,31,32^, stroke^33,34^, depression^35–38^, schizophrenia^39,40^, Alzheimer’s disease^41–43^, or to study changes in EEG complexity depending on age^44^, and under the influence of the external electromagnetic field^45^. HFD can be used also for the analysis of other biomedical signals, for example time series obtained from functional MRI^46–48^.

A first step towards functional brain mapping using the HFD method was limited to only two brain regions: the primary somatosensory and motor areas using scalp EEG. In this study, we aimed to extend this approach and to create a complete functional brain atlas using this measure with the intracerebral data obtained from other regions of the brain^49,50^.

Given the disadvantages of scalp EEG, use of HFD in direct cortical recordings offers the unique opportunity to not only have high temporal but also high spatial resolution and to investigate the local behavior of various brain structures regarding signal complexity across the different stages of vigilance. In particular, iEEG allows to investigate the nonlinear characteristics of the deep brain structures using the HFD of the iEEG data. In the current study the HFD method was applied to iEEG data during sleep provided by the Montreal Neurological Institute and Hospital, the Centre Hospitalier de l’Université de Montreal, and Grenoble-Alpes University Hospital (https://mni-open-ieegatlas.research.mcgill.ca/). ^11–13^.

We hypothesized that the signal complexity will decrease when sleep becomes deeper, that these changes will be region-specific, and that the behavior of some areas will show different signatures depending on the level of vigilance.

## Materials and methods

### Data acquisition

**Figure 1:**
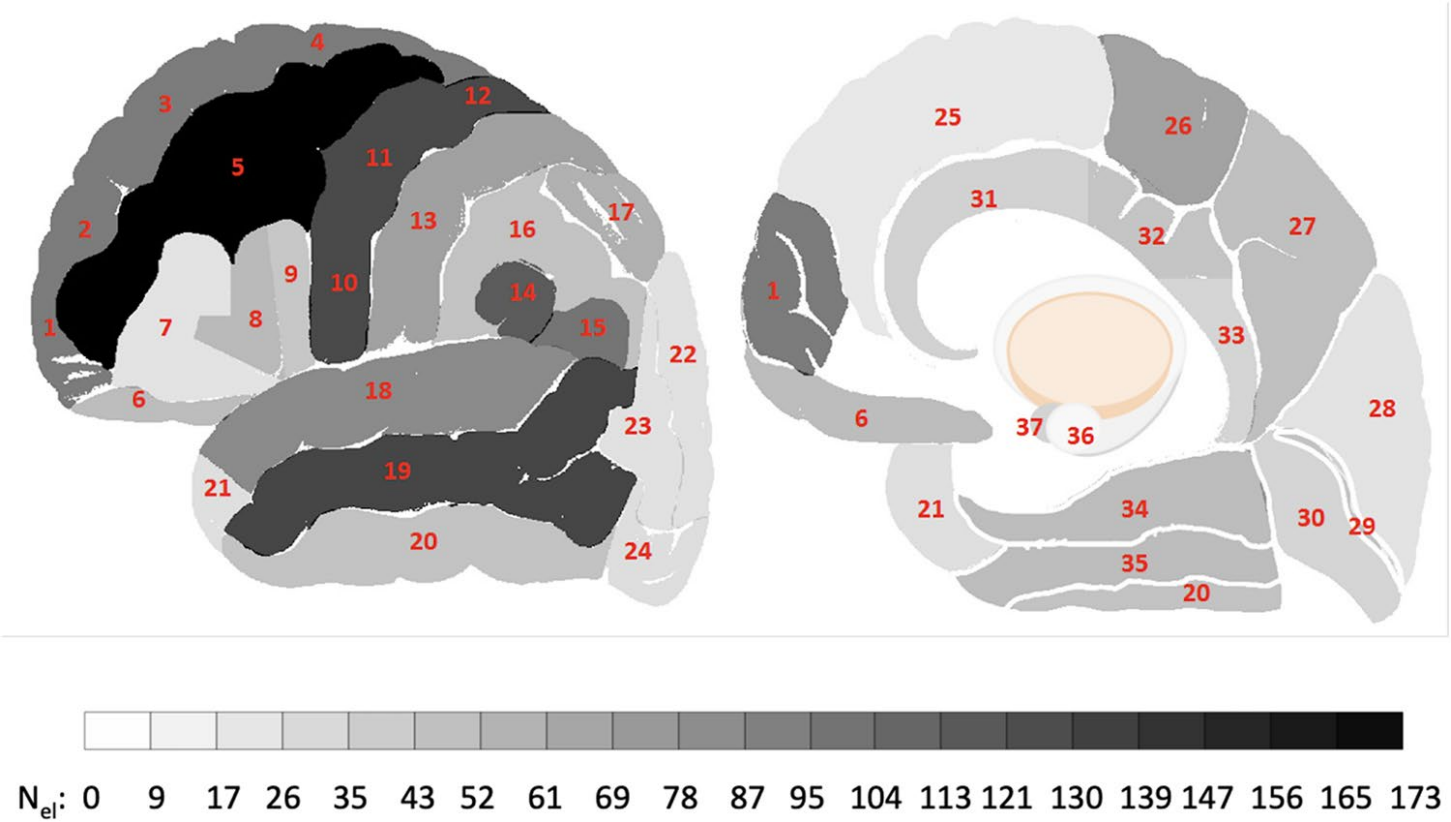
Illustration of the electrode coverage in the investigated human brain areas. The gray scale shows the number of electrodes (N_el_) placed in the respective brain region. The following brain areas are marked in the lateral view (left scheme) and in the medial view (right scheme): 1. Frontal pole; 2–4. Superior frontal gyrus (superior, middle and inferior segments); 5. Middle frontal gyrus; 6. Gyrus rectus and orbital gyri; 7–9. Inferior frontal gyrus (superior, middle and inferior segments); 10–12. Precentral gyrus (inferior, middle and superior segments); 13. Postcentral gyrus; 14. Supramarginal gyrus; 15. Angular gyrus; 16. Inferior parietal lobule; 17. Superior parietal lobule; 18–20. Temporal gyrus (superior, middle and inferior segments); 21. Temporal pole; 22–24. Occipital gyrus (superior, middle and inferior segments); 25. Medial frontal cortex; 26. Paracentral lobule; 27. Precuneus; 28. Cuneus; 29. Calcarine sulcus; 30. Lingual gyrus; 31–33. Anterior, middle and posterior cingulate gyrus; 34. Parahippocampal gyrus; 35. Fusiform gyrus; 36. Hippocampus; 37. Amygdala.

iEEG data were recorded from 1520 channels with stereo-EEG electrodes and from 265 channels with cortical grids and strips from 38 brain regions (see Figs. 1 and 4 and Supplementary Table 1) during the awake state with eyes closed and during different sleep stages (R, N2 and N3) in 106 patients with drug-resistant focal epilepsy obtained from three centers: Montreal Neurological Institute and Hospital (MNI), Centre Hospitalier de l’Université de Montreal, and Grenoble-Alpes University Hospital. The database contains one-minute EEG signals recorded in four stages (wake, R, N2 and N3) from a total maximum number of 1772 bipolar channels with a sampling frequency of 200 Hz. For the purpose of this atlas, only channels from presumably normal brain regions were selected, comprising approximately 20% of all implanted electrode contacts^11,12^. The protocol for the study was approved by the MNI (REB vote: MUHC-15-950), and the informed consent was obtained from each subject. The data are publically available at the MNI website (https://mni-open-ieegatlas.research.mcgill.ca/). ^14^

### Higuchi fractal dimension method

The HFD measures the complexity of signal curves. This measure specifies the degree to which the curve covers the plane. It takes values from one for the Euclidean dimension of line to two for the dimension of plane. The more complex the curve (from line to white noise), the greater is the HFD value.

The HFD is calculated as the angular coefficient of the linear regression of the relationship between logarithm of length of the curve L and logarithm of the inverse of parameter k. The only parameter of HFD is a maximal k value. It is a limit value of k for which HFD does not change anymore.

In order to calculate the *k* values of *L* the algorithm constructs *k* new time series from a given signal *X(i), i* = *1,..,N* samples:

1 $$ X(m),X(m + k),...,X\left( {m + {\text{int}} \left( {\frac{N - m}{k}} \right) \cdot k} \right) $$

where *m* is an initial time changing from 1 to *k* ; int(r) is integer part of a real number r.

The length of each of m curves is calculated as:

2 $$ L_{m} = \frac{1}{k}\left[ {\,\mathop \Sigma \limits_{{i = 1,{\text{int}} \left( {\frac{N - m}{k}} \right)}} \;\left| {\,X(m + ik) - X(m + (i - 1)k)\,} \right| \cdot \,\,\frac{N - 1}{{{\text{int}} \left( {\frac{N - m}{k}} \right)}}\,} \right] $$

The length of curve *L* is calculated as the mean of the *k* values of *L*_*m*_.

Here, the HFD was calculated in one-second windows for the maximal k value equal to 16.

### Statistical analysis

A Wilcoxon test was performed to find statistically significant differences between each pair of the four brain states (wake, R, N2 and N3) at the significance level equal to 0.05. The Wilcoxon test is dedicated to test differences between dependent variables (here HFD from 38 brain regions), thus it is appropriate for taking into account the synchronous interaction between different brain regions (brain connectivity). While the lack of the correction for the 4 sleep stages stems from the assumption that the EEG signal is stable at different stages of sleep occurring 4–5 times in a cyclic manner throughout the entire sleep.

The nonlinearity of the EEG signals was tested using surrogate data. The surrogate data were calculated using the surrogate function implemented in the WaveForm DataBase (WFDB) Toolbox in Matlab^51^. Then, the Wilcoxon signed rank test was performed using *signrank* function from the Statistics and Machine Learning Toolbox in Matlab to test the null hypothesis that the difference between the HFD of the original data and the HFD of the surrogate data come from a distribution whose median is zero at the significance level equal to 0.05. The results showed that only one percent of all EEG segments were linear in stages W, R and N2, while in stage N3 all segments were nonlinear.

### Ethics statement

This study was approved by the Research Ethics Board of the Montreal Neurological Institute and Hospital. All patients signed a Research Ethics Board approved written informed consent. The research was performed in accordance with the Declaration of Helsinki.

## Results

### Dependence of EEG complexity on the vigilance state and topography

**Figure 2:**
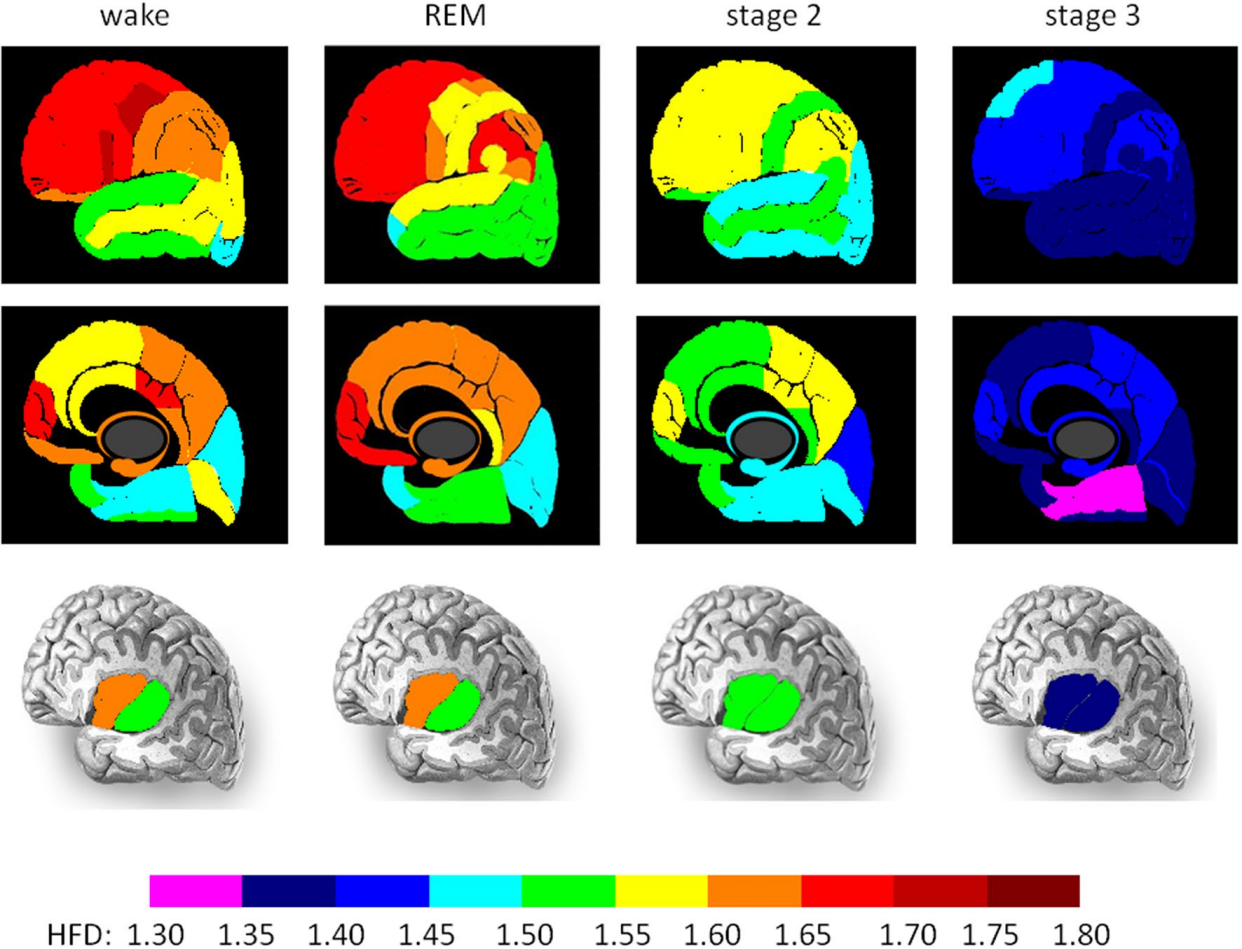
Higuchi Fractal Dimension (HFD) of different brain areas in four sleep stages (wake, R, stage N2 and stage N3). The results are shown on lateral, medial and insular views (from the upper to the lower row). The anterior and posterior parts of insula are illustrated separately. A graduate decrease of HFD from wake to stage N3 can be observed (from marron/red to blue/lilac colour on the scale). The highest HFD is observed in the frontal lobe during the awake state and stage R (red areas). The lowest HFD was in parahippocampal and fusiform gyri during stage N3 (lilac colour).

**Figure 3:**
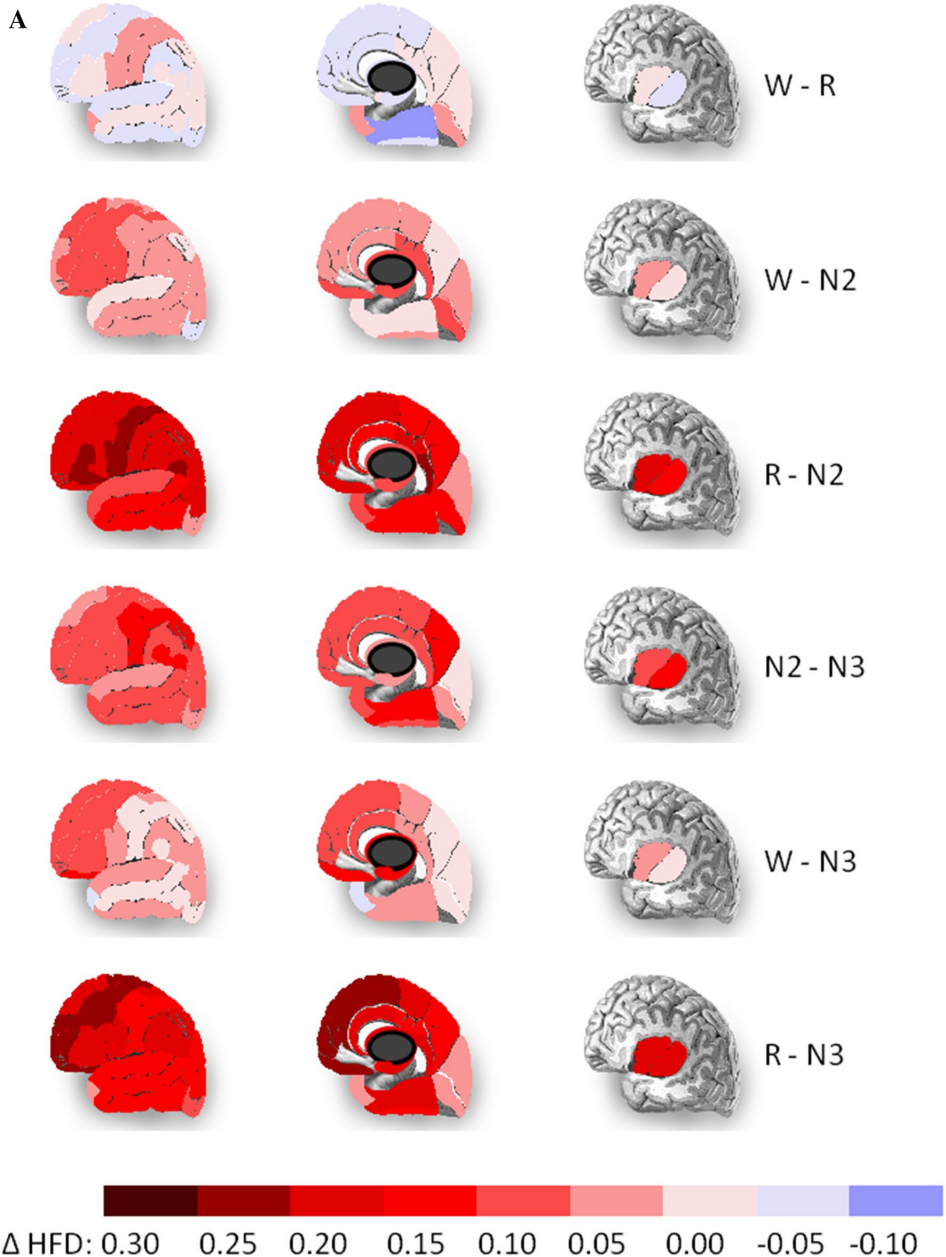

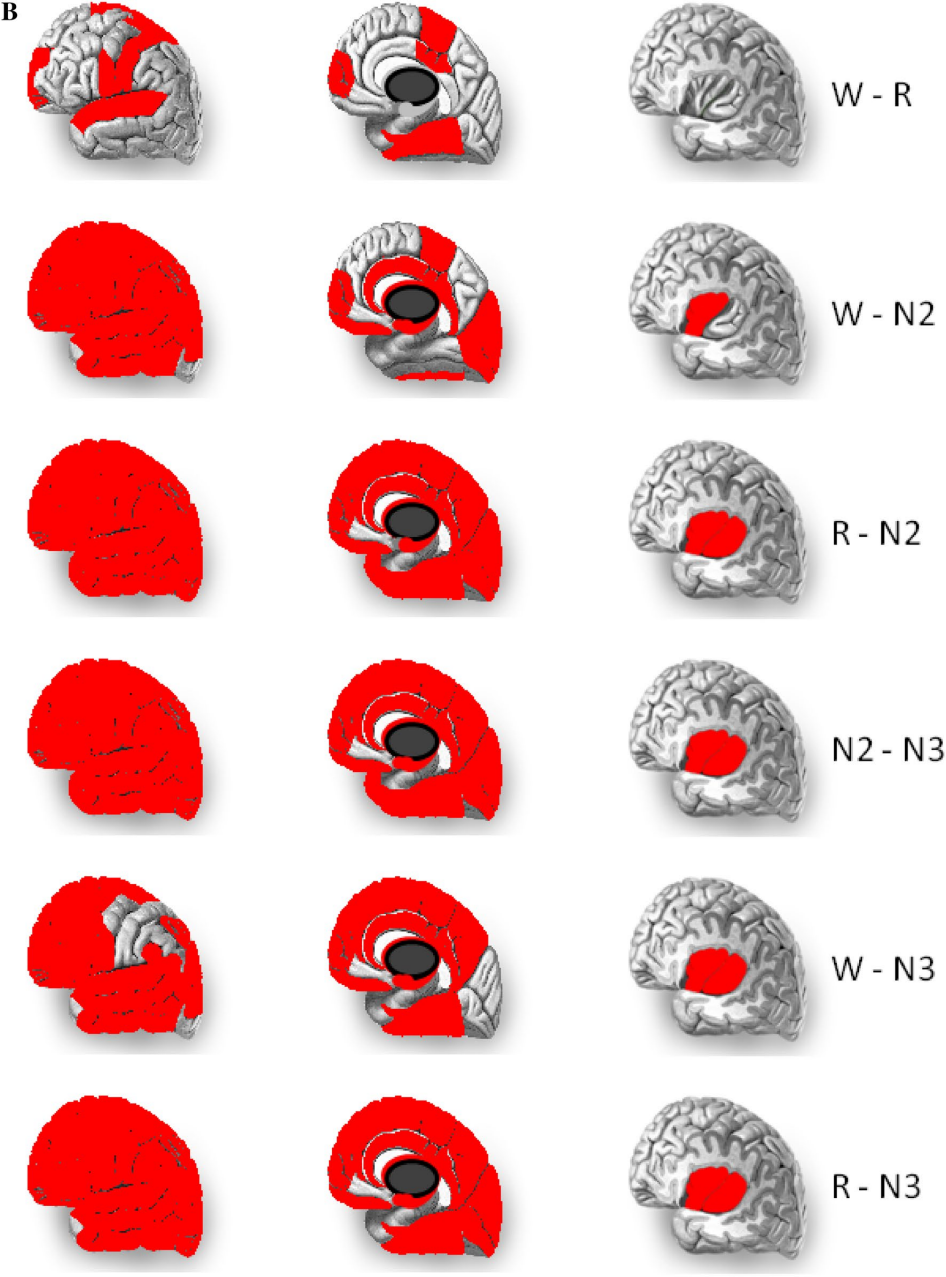
Topographical differences between each pair of sleep stages. The results are shown on lateral, medial and insular views (from the left to the right column). The anterior and posterior parts of insula are illustrated separately. (**A**) The differences between HFD of two vigilance stages. (**B**) Areas for which differences between two vigilance stages were significant (marked in red). All statistically significant differences between the HFD values in two stages were positive.

The complexity of EEG signals depends on the vigilance state. The signal is most complex during wakefulness (first column in Fig. 2) and least complex during stage N3 (last column in Fig. 2 and 5th row in Fig. 3A). REM in contrast to non-REM sleep is characterized by a higher EEG complexity with HFD values similar to those during the awake state (second column in Fig. 2, and 1st and 6th row in Fig. 3A). There are marked differences in HFD values between regions. The highest EEG complexity is observed in the frontal cortex (HFD greater than 1.65 ± 0.1; see the areas marked in red in Fig. 2). Two areas are particularly active during wakefulness: the middle segment of the precentral gyrus (area 11 in Fig. 1) and the inferior segment of the inferior frontal gyrus (area 9 in Fig. 1). Both areas are characterized by a HFD greater than 1.7 ± 0.1. The HFD gradually decreases in stages N2 and N3 in comparison to awake and R stages. In stage N2 the occipital lobe (cuneus, calcarine sulcus and lingual gyrus) has the lowest HFD, i.e., values from 1.4 ± 0.1 to 1.45 ± 0.1 (see blue areas in the medial view on Fig. 2). During stage N3 two other areas, parahippocampal gyrus and fusiform gyrus, are characterized by the lowest complexity, i.e., HFD reaching values less than 1.35 ± 0.1 (see the areas marked with lilac colour in the medial view on Fig. 2).

### Topographical differences between sleep stages

**Figure 4:**
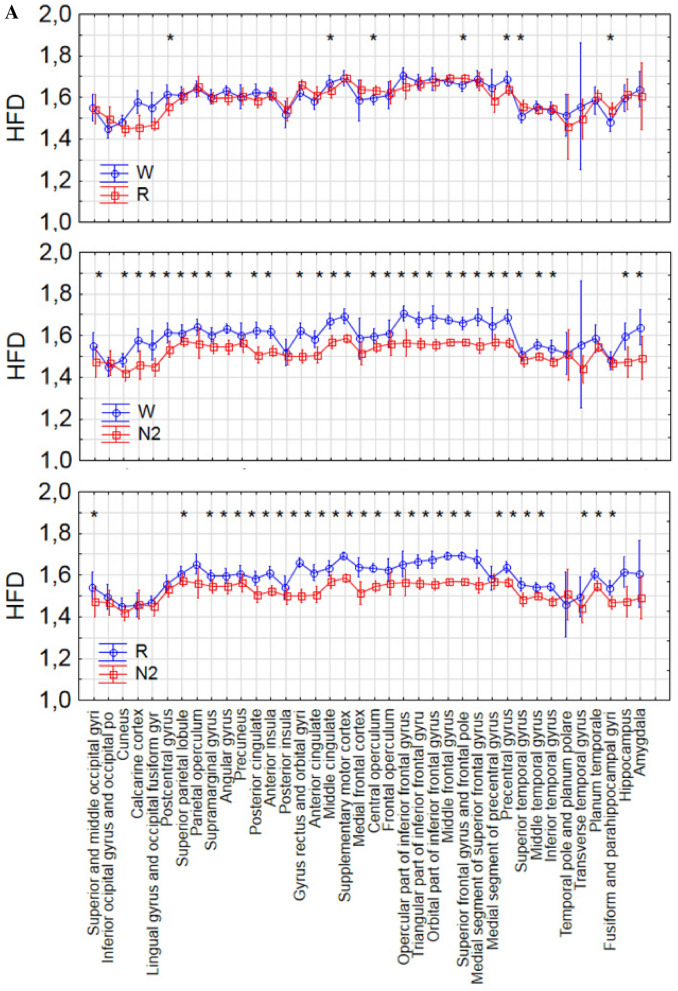

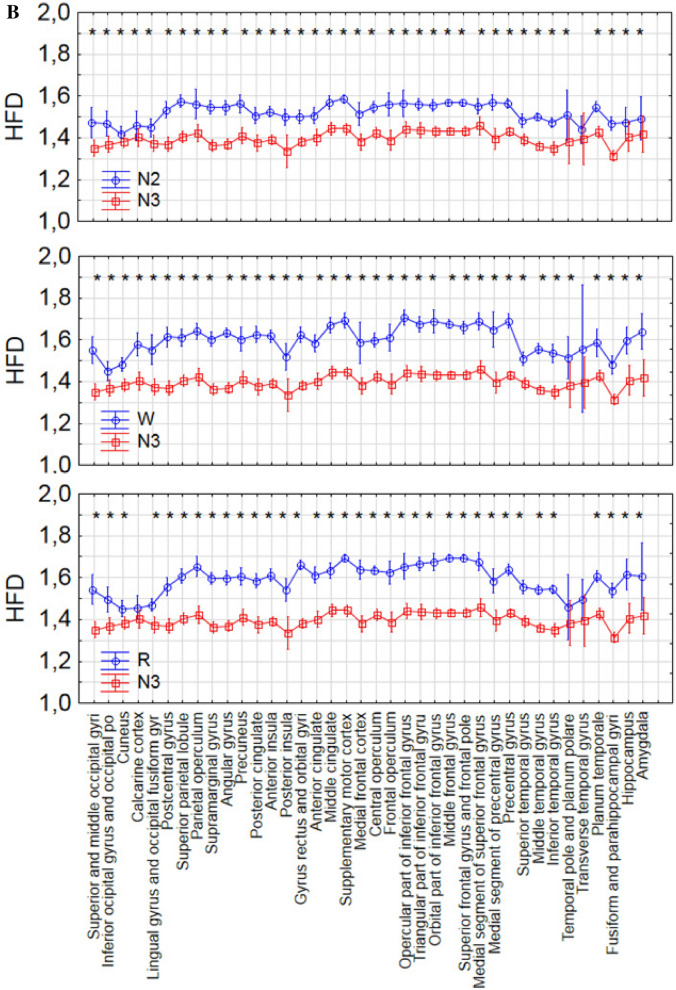
The differences between each pair of sleep stages in 38 brain areas. The statistically significant differences were marked with asterisks.

Statistically significant differences were found between every pair of sleep stages (Figs. 3, 4 and Supplementary Table 1). Signal complexity was more different between N2 and N3 sleep as opposed to between the awake state and N2 or stage R and N2 sleep (difference in 99.2% of electrodes vs. 86.2% vs. 82.7%). The results of the Wilcoxon test show that the occipital (regions 22–24 and 28–30 in Fig. 1) and temporal regions (regions 18–21 and 34–35 in Fig. 1) behave differently from the frontal (regions 1–9 and 25, in Fig. 1) and central regions (regions 10–13 and 26 in Fig. 1).

### REM vs. wake

The largest difference between the stages R and wake was in the EEG complexity in the precentral gyrus (p-value = 0.002, Z = 3.06; see the area marked with red in the first upper row on Fig. 3). Significant differences were found also in the superior temporal gyrus (p-value = 0.005, Z = 3.47), fusiform and parahippocampal gyri (p-value = 0.005, Z = 2.82), postcentral gyrus (p-value = 0.03, Z = 2.21), middle cingulate (p-value = 0.04, Z = 2.07), central operculum (p-value = 0.03, Z = 2.13), superior frontal gyrus and frontal pole (p-value = 0.02, Z = 2.37).

### REM vs. N2 and wake vs. N2

Most of the brain differs significantly when comparing REM with N2 and wake with N2, but for several brain areas the complexity does not change. These areas are located mainly in the occipital and temporal part of brain, i.e. inferior occipital gyrus and occipital pole, temporal pole and planum polare, transverse temporal gyrus (Heschl’s gyrus).

In some areas the HFD during stage N2 is not different from the HFD during wakefulness, although it differs from the HFD during stage R, and vice versa. No differences were found between the awake stage and stage N2 in the precuneus, posterior insula, and medial frontal cortex, parahippocampal and fusiform gyri, while the EEG complexity in these areas was different comparing stages R and N2 (Fig. 4 and Supplementary Table 1). However, R and N2 had a similar HFD in other areas such as the cuneus, calcarine cortex, lingual gyrus and occipital fusiform gyrus, medial segment of precentral gyrus, amygdala, postcentral gyrus and parietal operculum. These areas had different HFD in wakefulness and N2.

### REM vs. N3

The vast majority (35 of 38 regions) of regions showed statistically significant differences between stages N2, N3 and R. No changes were found in the calcarine sulcus, temporal pole and planum polare, and transverse temporal gyrus (Fig. 4 and Supplementary Table 1).

### Similar complexity in all vigilance stages

The transverse temporal gyrus (Heschl’s gyrus) was the only structure, where no statistically significant differences in signal complexity were found between any two vigilance stages (Supplementary Table 1).

## Discussion

The complexity analysis of a unique database of iEEG signals recorded from a large number of brain areas (38 regions) in 106 human subjects allowed to create a functional brain atlas for the awake and sleeping human brain. The comparison between signal complexity for each pair of brain states has led to novel and interesting findings. We found that (i) despite a global increase in EEG complexity in the more alert brain, several brain structures do not follow this general tendency, but reveal specific fingerprints of EEG complexity across the different states of vigilance, (ii) the occipital lobe and neighbouring posterior cortical regions behave differently compared to anterior brain regions, and show similar complexity levels across the sleep–wake-cycle, and (iii) there are large differences between the two stages of NREM sleep N2 and N3 which may be related to the appearance of delta waves in stage N3 because HFD has lower values for lower frequencies.

### Specific topographic fingerprints of brain complexity

The precentral gyrus was the main structure that showed a difference in complexity as assessed with the HDF between the awake state and REM sleep. This is interesting, as the precentral gyrus is a motor function-related area characterized by a clear peak in the beta band during wakefulness^11^ and in the theta band during stage R^13^. This area plays an important role in sensorimotor integration^52^. Lesions in this area result in motor deficits. This result is consistent with one of the hallmarks of stage R, which is temporary muscle atonia. Another study showed that this region is the only one showing significant activation prior to K-complexes with arousals as opposed to those not being followed by arousals, which would mean that if this region shows more wake-similar activity people are more likely to arouse after a K-complex^53^. Significant differences were also present in other areas located mainly in temporal lobe: superior temporal gyrus, fusiform and parahippocampal gyri.

Another interesting finding is that the transverse temporal gyrus, which is the main structure responsible for processing of auditory information**,** does not show any difference in complexity between any sleep stages. These results confirm functional MRI studies which showed inhibition of the response of auditory and visual cortices on external stimuli during transition from wake to sleep^54,55^. The HFD values for R and N2 are closer to the values for N3 than for wake. It could therefore be argued that these regions go into deeper sleep earlier, maybe to facilitate sleep, as reflected in the inhibition of auditory responses. Apart of the transverse temporal gyrus, no differences in HFD were found between deep sleep (stage N3) and N2 predominantly in two regions: the calcarine sulcus, which divides the visual cortex in two parts: cuneus, and fusiform and lingual gyri, and the temporal pole and planum polare (see Fig. 4 and Supplementary Table 1). The role of this area has yet to be clarified. It is considered to be part of the limbic system involved in social and emotional processing, including face recognition and mind theory^56^. Another study showed that one night of acute sleep deprivation causes a significant decrease of the gray matter volume and cortical thickness of the right temporal pole, which is associated with personal and episodic memory^57^. Considering this crucial role of slow wave sleep for brain health, the temporal pole seems to be an interesting subject of further research.

The hippocampus showed no difference in the HFD between the awake state and stage R, while the fusiform and parahippocampal gyri were the only areas that showed no difference between the awake state and N2 (Fig. 4 and Supplementary Table 1). Moreover, similarity in HFD was observed between stages R and N2 only for the postcentral gyrus, the area containing the primary somatosensory cortex (Fig. 4 and Supplementary Table 1). Recently, it was shown that both spontaneous and periphery evoked spindles are generated in the primary somatosensory cortex located in the postcentral gyrus^58,59^. Moreover, another recent study showed a role of spindles in the onset of stage R of sleep. Their rate increased before the onset of stage R, but not wakefulness^60^. Sleep spindles are bursts of higher frequency (11–15 Hz) emerging from the lower frequency background. Generally, the HFD is lower during stage 2 than R stage. However, the HFD increases for higher frequencies. Thus, the contribution of sleep spindles may explain the increase of the HFD to the level characteristic for R stage in spindle generating regions.

### Stable sleep complexity of the occipital and temporal cortex

The complexity did not change from wake (or stage R) to stage N2 mainly in areas located in the occipital and temporal part of brain, i.e. i.e. inferior occipital gyrus and occipital pole, temporal pole and planum polare, transverse temporal gyrus (Heschl’s gyrus). The HFD in stages N2 and N3 were similar only in transverse temporal gyrus. The same region had similar HFD values in all sleep stages (N2, N3 and R). These findings add to the evidence indicating that the occipital and temporal cortex is less affected by the marked changes that characterize the different sleep stages in fronto-central head regions.

### Stage-specific differences in complexity

The results of our study showed differences between regions in each of the four vigilance stages as well as differences between each pair of these stages. This single measure reveals new aspects of sleep dynamics, particularly important regional differences. There is a global tendency of less complexity in the signal the deeper the stage of sleep, with different brain structures showing particular differences or none across sleep stages. The first finding confirmed results from our previous studies using scalp EEG as global measure of brain activity that the HFD decreased gradually passing through the successive sleep stages from wake to deep sleep and increased again during stage R in most areas of the brain^21,22,25^. The latter finding might be related to the specific function of different brain structures for sleep. This supports the hypothesis of local sleep governed by global input from the thalamus and brain stem structures as evident by global changes across the different states of vigilance^59,61,62^. In line with the results of spectral analysis performed using the same data^11,13^, the highest HFD values (from 1.65 to 1.8) during the awake state in the frontal lobe correspond to the higher frequencies (beta and gamma) activity in this region. The HFD reached the maximal value of 1.8 in the medial segments of precentral and inferior frontal gyri, where significant peaks in the beta frequency band were present in most channels^11^. The lowest HFD values (from 1.45 to 1.5) were found in the occipital lobe, and particularly in the cuneus, inferior occipital gyrus and occipital pole, all regions having significant peaks in lower frequencies (delta, theta and lower alpha)^11^. In line with the results of spectral analysis, the EEG complexity revealed differences between N2 and N3 sleep in the vast majority (99.2%) of electrodes, which may be related with the occurrence of lower frequencies (delta waves) in N3 what causes a decrease of HFD values.

## Conclusion and future prospects

The atlas of brain complexity in different stages of vigilance based on a large iEEG dataset allowed to advance the current state-of-art by providing new insights into the mechanisms of sleep generation. More specifically, it allowed us to discover that the traditional sleep stages are clearly differentiated in fronto-central brain regions but that temporo-parieto-occipital regions sleep differently, to the point that the transverse temporal gyrus does not show differentiated sleep stages; also that the main difference between wakefulness and R was in primary motor region. The atlas of brain complexity in different stages of vigilance in healthy sleep may be helpful in identification of specific brain areas responsible for various sleep disorders. A decrease of complexity in relation to its value in norm may be a good marker of deterioration of specific brain area's function. We have found several regions that sleep differently from the rest of the brain. Further studies are warranted to deepen our understanding of the role of the regions that sleep differently from the rest of the brain, and their involvement in various sleep disorders compatible with an impaired sleep–wake boundary control. Moreover, the dependence of HFD on parameters and bifurcations in models should be investigated to understand better the mechanisms of sleep generation.

## Supplementary Information

Supplementary Information.

## Data Availability

The data are publically available at the MNI website (https://mni-open-ieegatlas.research.mcgill.ca/). Figures 1–3 were generated using Matlab function SleepAtlas available at the IBEB PAS website (https://ibib.waw.pl/download/EO/software.zip).
