# Supplementary material for: Region-specific complexity of the intracranial EEG in the sleeping human brain
Source: Sci Rep. 2022 Jan 10;12:451. doi: 10.1038/s41598-021-04213-8 (PMC8748934; doi:10.1038/s41598-021-04213-8)
Supplement: Supplementary file 1 — Supplementary Information. [file 41598_2021_4213_MOESM1_ESM.pdf]

brain regions	L	M	N channels	wake - R	wake – N2	wake– N3	R– N2	R – N3	N2 – N3
Superior and middle occipital gyri	22,23		21	p=0.43; Z=0.78 HFD=1.53±0.10 vs. 1.54±0.12	p=0.001; Z=3.30 HFD=1.53±0.10 vs. 1.48±0.12	P=0.001; Z=3.30 HFD=1.53±0.10 vs. 1.35±0.06	p=0.001; Z=3.30 HFD=1.54±0.12 vs. 1.48±0.12	p=0.001; Z=3.30 HFD=1.54±0.12 vs. 1.35±0.06	p=0.001; Z=3.30 HFD=1.48±0.12 vs. 1.35±0.06
Inferior ocipital gyrus and occipital pole	24		23	p=0.10; Z=1.63 HFD=1.47±0.09 vs. 1.50±0.12	p=0.43; Z=0.78 HFD=1.47±0.09 vs. 1.46±0.10	p=0.0005; Z=3.48 HFD=1.47±0.09 vs. 1.36±0.07	p=0.10; Z=1.63 HFD=1.50±0.12 vs. 1.46±0.10	p=0.0004; Z=3.57 HFD=1.50±0.12 vs. 1.36±0.07	p=0.0003; Z=3.62 HFD=1.46±0.10 vs. 1.36±0.07
Cuneus		28	19	p=0.11; Z=1.59 HFD=1.49±0.06 vs. 1.45±0.08	p=0.014; Z=2.46 HFD=1.49±0.06 vs. 1.42±0.08	p=0.0003; Z=3.59 HFD=1.49±0.06 vs. 1.38±0.06	p=0.06; Z=1.85 HFD=1.45±0.08 vs. 1.42±0.08	p=0.0003; Z=3.64 HFD=1.45±0.08 vs. 1.38±0.06	p=0.003; Z=2.94 HFD=1.42±0.08 vs. 1.38±0.06
Calcarine cortex (primary visual cortex)		29	12	p=0.05; Z=1.96 HFD=1.57±0.07 vs. 1.46±0.07	p=0.04; Z=2.10 HFD=1.57±0.07 vs. 1.43±0.09	p=0.01; Z=2.52 HFD=1.57±0.07 vs. 1.38±0.07	p=0.78; Z=0.28 HFD=1.46±0.07 vs. 1.43±0.09	p=0.09; Z=1.68 HFD=1.46±0.07 vs. 1.38±0.07	p=0.04; Z=2.10 HFD=1.43±0.09 vs. 1.38±0.07
Lingual gyrus and occipital fusiform gyrus		30	29	p=0.05; Z=1.96 HFD=1.54±0.13 vs. 1.47±0.05	p=0.012; Z=2.51 HFD=1.54±0.13 vs. 1.44±0.06	p=0.003; Z=2.98 HFD=1.54±0.13 vs. 1.37±0.07	p=0.16; Z=1.42 HFD=1.47±0.05 vs. 1.44±0.06	p=0.003; Z=2.98 HFD=1.47±0.05 vs. 1.37±0.07	p=0.005; Z=2.82 HFD=1.44±0.06 vs. 1.37±0.07
Postcentral gyrus (including medial segment)	13		64	p=0.03; Z=2.21 HFD=1.63±0.14 vs. 1.56±0.12	p=0.0004; Z=3.57 HFD=1.63±0.14 vs. 1.52±0.11	p=0.000002; Z=4.80 HFD=1.63±0.14 vs. 1.37±0.10	p=0.08; Z=1.74 HFD=1.56±0.12 vs. 1.52±0.11	p=0.000001; Z=4.82 HFD=1.56±0.12 vs. 1.37±0.10	p=0.000001; Z=4.86 HFD=1.52±0.11 vs. 1.37±0.10
Superior parietal lobule	17		53	p=0.94; Z=0.08 HFD=1.59±0.12 vs. 1.61±0.10	p=0.04; Z=2.01 HFD=1.59±0.12 vs. 1.57±0.09	p=<0.000001; Z=5.18 HFD=1.59±0.12 vs. 1.41±0.10	p=0.0014; Z=3.19 HFD=1.61±0.10 vs. 1.57±0.09	p=<0.000001; Z=5.23 HFD=1.61±0.10 vs. 1.41±0.10	p<0.000001; Z=5.23 HFD=1.57±0.09 vs. 1.41±0.10
Parietal operculum	16		41	p=0.58; Z=0.56 HFD=1.63±0.13 vs. 1.65±0.07	p=0.02; Z=2.29 HFD=1.63±0.13 vs. 1.55±0.08	p=0.005; Z=2.80 HFD=1.63±0.13 vs. 1.43±0.08	p=0.07; Z=1.78 HFD=1.65±0.07 vs. 1.55±0.08	p=0.005; Z=2.80 HFD=1.65±0.07 vs. 1.43±0.08	p=0.005; Z=2.80 HFD=1.55±0.08 vs. 1.43±0.08
Supramarginal gyrus	14		70	p=0.44; Z=0.77 HFD=1.64±0.12 vs. 1.59±0.09	p=0.0001; Z=3.82 HFD=1.64±0.12 vs. 1.55±0.09	p=<0.000001; Z=5.44 HFD=1.64±0.12 vs. 1.40±0.12	p=0.0001; Z=3.81 HFD=1.59±0.09 vs. 1.55±0.09	p=<0.000001; Z=5.44 HFD=1.59±0.09 vs. 1.40±0.12	p=<0.000001; Z=5.44 HFD=1.55±0.09 vs. 1.40±0.12
Angular gyrus	15		53	p=0.07; Z=1.79 HFD=1.62±0.09 vs. 1.60±0.11	p=0.000002; Z=4.81 HFD=1.62±0.09 vs. 1.54±0.10	p=<0.000001; Z=5.65 HFD=1.62±0.09 vs. 1.38±0.09	p=0.00004; Z=4.11 HFD=1.60±0.11 vs. 1.54±0.10	p<0.000001; Z=5.65 HFD=1.60±0.11 vs. 1.38±0.09	p<0.000001; Z=5.65 HFD=1.54±0.10 vs. 1.38±0.09
Precuneus		27	43	p=0.44; Z=0.77 HFD=1.61±0.15 vs. 1.60±0.11	p=0.07; Z=1.83 HFD=1.61±0.15 vs. 1.56±0.12	p=0.000001; Z=4.84 HFD=1.61±0.15 vs. 1.41±0.11	p=0.0013; Z=3.22 HFD=1.60±0.11 vs. 1.56±0.12	p=0.000001; Z=4.94 HFD=1.60±0.11 vs. 1.41±0.11	p=0.000001; Z=4.8 HFD=1.56±0.12 vs. 1.41±0.11
Posterior cingulate		33	29	p=0.08; Z=1.73 HFD=1.67±0.09 vs. 1.58±0.06	p=0.001; Z=3.30 HFD=1.67±0.09 vs. 1.53±0.09	p=0.001; Z=3.30 HFD=1.67±0.09 vs. 1.40±0.09	p=0.002; Z=3.04 HFD=1.58±0.06 vs. 1.53±0.09	p=0.001; Z=3.30 HFD=1.58±0.06 vs. 1.40±0.09	p=0.001; Z=3.30 HFD=1.53±0.09 vs. 1.40±0.09
Anterior insula			71	p=0.76; Z=0.31 HFD=1.65±0.12 vs. 1.61±0.09	p=0.000003; Z=4.70 HFD=1.65±0.12 vs. 1.54±0.09	p<0.000001; Z=5.16 HFD=1.65±0.12 vs. 1.40±0.07	p=0.000001; Z=4.82 HFD=1.61±0.09 vs. 1.54±0.09	p<0.000001; Z=5.16 HFD=1.61±0.09 vs. 1.40±0.07	p<0.000001; Z=5.16 HFD=1.54±0.09 vs. 1.40±0.07
Posterior insula			35	p=0.25; Z=1.61 HFD=1.59±0.14 vs. 1.54±0.10	p=0.47; Z=0.72 HFD=1.59±0.14 vs. 1.52±0.08	p=0.0012; Z=3.23 HFD=1.59±0.14 vs. 1.39±0.13	p=0.0132; Z=2.48 HFD=1.54±0.10 vs. 1.52±0.08	p=0.001; Z=3.30 HFD=1.54±0.10 vs. 1.39±0.13	p=0.001; Z=3.30 HFD=1.52±0.08 vs. 1.39±0.13
Gyrus rectus and orbital gyri	6	6	45	p=0.07; Z=1.84 HFD=1.64±0.10 vs. 1.66±0.06	p=0.00002; Z=4.28 HFD=1.64±0.10 vs. 1.52±0.08	p=0.000004; Z=4.62 HFD=1.64±0.10 vs. 1.40±0.08	p=0.000004; Z=4.62 HFD=1.66±0.06 vs. 1.52±0.08	p=0.000004; Z=4.62 HFD=1.66±0.06 vs. 1.40±0.08	p=0.000004; Z=4.62 HFD=1.52±0.08 vs. 1.40±0.08

brain regions	L	M	N channels	wake - R	wake – N2	wake– N3	R – N2	R – N3	N2 – N3
Anterior cingulate		31	31	p=0.26; Z=1.13 HFD=1.59±0.08 vs. 1.61±0.08	p=0.01; Z=2.50 HFD=1.59±0.08 vs. 1.52±0.07	p=0.0002; Z=3.78 HFD=1.59±0.08 vs. 1.42±0.08	p=0.0003; Z=3.66 HFD=1.61±0.08 vs. 1.52±0.07	p=0.0001; Z=3.82 HFD=1.61±0.08 vs. 1.42±0.08	p=0.0002; Z=3.70 HFD=1.52±0.07 vs. 1.42±0.08
Middle cingulate		32	40	p=0.04; Z=2.07 HFD=1.65±0.12 vs. 1.63±0.08	p=0.0002; Z=3.70 HFD=1.65±0.12 vs. 1.57±0.08	p=0.00006; Z=4.01 HFD=1.65±0.12 vs. 1.47±0.07	p=0.0002; Z=3.70 HFD=1.63±0.08 vs. 1.57±0.08	p=0.00006; Z=4.01 HFD=1.63±0.08 vs. 1.47±0.07	p=0.00006; Z=4.01 HFD=1.57±0.08 vs. 1.47±0.07
Supplementary motor cortex			47	p=0.39; Z=0.87 HFD=1.67±0.09 vs. 1.69±0.05	p=0.00004; Z=4.10 HFD=1.67±0.09 vs. 1.58±0.06	p=0.00004; Z=4.62 HFD=1.67±0.09 vs. 1.45±0.07	p=0.000005; Z=4.58 HFD=1.69±0.05 vs. 1.58±0.06	p=0.000004; Z=4.62 HFD=1.69±0.05 vs. 1.45±0.07	p=0.000004; Z=4.62 HFD=1.58±0.06 vs. 1.45±0.07
Medial frontal cortex		25	19	p=0.20; Z=1.27 HFD=1.63±0.12 vs. 1.64±0.06	p=0.17; Z=1.38 HFD=1.63±0.12 vs. 1.52±0.06	p=0.005; Z=2.80 HFD=1.63±0.12 vs. 1.38±0.05	p=0.005; Z=2.80 HFD=1.64±0.06 vs. 1.52±0.06	p=0.005; Z=2.80 HFD=1.64±0.06 vs. 1.38±0.05	p=0.005; Z=2.80 HFD=1.52±0.06 vs. 1.38±0.05
Central operculum		26	63	p=0.03; Z=2.13 HFD=1.62±0.11 vs. 1.64±0.06	p=0.001; Z=3.30 HFD=1.62±0.11 vs. 1.54±0.07	p=0.000003; Z=4.70 HFD=1.62±0.11 vs. 1.43±0.08	p=0.000009; Z=4.44 HFD=1.64±0.06 vs. 1.54±0.07	p=0.000003; Z=4.70 HFD=1.64±0.06 vs. 1.43±0.08	p=0.000003; Z=4.70 HFD=1.54±0.07 vs. 1.43±0.08
Frontal operculum			29	p=0.74; Z=0.33 HFD=1.64±0.13 vs. 1.62±0.11	p=0.012; Z=2.50 HFD=1.64±0.13 vs. 1.56±0.10	p=0.0002; Z=3.72 HFD=1.64±0.13 vs. 1.40±0.09	p=0.03; Z=2.11 HFD=1.62±0.11 vs. 1.56±0.10	p=0.0002; Z=3.72 HFD=1.62±0.11 vs. 1.40±0.09	p=0.0002; Z=3.72 HFD=1.56±0.10 vs. 1.40±0.09
Opercular part of inferior frontal gyrus	9		38	p=0.06; Z=1.90 HFD=1.71±0.07 vs. 1.65±0.13	p=0.0003; Z=3.62 HFD=1.71±0.07 vs. 1.59±0.12	p=0.00009; Z=3.92 HFD=1.71±0.07 vs. 1.46±0.09	p=0.00009; Z=3.92 HFD=1.65±0.13 vs. 1.59±0.12	p=0.0001; Z=3.88 HFD=1.65±0.13 vs. 1.46±0.09	p=0.0002; Z=3.70 HFD=1.59±0.12 vs. 1.46±0.09
Triangular part of inferior frontal gyrus	8		47	p=0.28; Z=1.07 HFD=1.66±0.10 vs. 1.66±0.09	p=0.0003; Z=3.64 HFD=1.66±0.10 vs. 1.56±0.07	p=0.000007; Z=4.49 HFD=1.66±0.10 vs. 1.46±0.11	p=0.00002; Z=4.25 HFD=1.66±0.09 vs. 1.56±0.07	p=0.000003; Z=4.70 HFD=1.66±0.09 vs. 1.46±0.11	p=0.000003; Z=4.70 HFD=1.56±0.07 vs. 1.46±0.11
Orbital part of inferior frontal gyrus	7		19	p=0.35; Z=0.94 HFD=1.66±0.09 vs. 1.68±0.07	p=0.0015; Z=3.18 HFD=1.66±0.09 vs. 1.55±0.04	p=0.0015; Z=3.18 HFD=1.66±0.09 vs. 1.44±0.05	p=0.0015; Z=3.18 HFD=1.68±0.07 vs. 1.55±0.04	p=0.0015; Z=3.18 HFD=1.68±0.07 vs. 1.44±0.05	p=0.0015; Z=3.18 HFD=1.55±0.04 vs. 1.44±0.05
Middle frontal gyrus	5		173	p=0.21; Z=1.24 HFD=1.69±0.10 vs. 1.69±0.10	p<0.000001; Z=7.49 HFD=1.69±0.10 vs. 1.58±0.08	p<0.000001; Z=8.94 HFD=1.69±0.10 vs. 1.45±0.09	p<0.000001; Z=8.80 HFD=1.69±0.10 vs. 1.58±0.08	p<0.000001; Z=8.94 HFD=1.69±0.10 vs. 1.45±0.09	p<0.000001; Z=8.93 HFD=1.58±0.08 vs. 1.45±0.09
Superior frontal gyrus and frontal pole	1,2,4	1	89	p=0.02; Z=2.37 HFD=1.65±0.11 vs. 1.69±0.07	p<0.000001; Z=5.69 HFD=1.65±0.11 vs. 1.56±0.08	p<0.000001; Z=6.95 HFD=1.65±0.11 vs. 1.43±0.09	p<0.000001; Z=6.95 HFD=1.69±0.07 vs. 1.56±0.08	p<0.000001; Z=6.96 HFD=1.69±0.07 vs. 1.43±0.09	p<0.000001; Z=6.92 HFD=1.56±0.08 vs. 1.43±0.09
Medial segment of superior frontal gyrus	3	3	16	p=0.22; Z=1.22 HFD=1.68±0.06 vs. 1.68±0.08	p=0.0015; Z=3.18 HFD=1.68±0.06 vs. 1.55±0.05	p=0.0015; Z=3.18 HFD=1.68±0.06 vs. 1.46±0.07	p=0.0015; Z=3.18 HFD=1.68±0.08 vs. 1.55±0.05	p=0.0015; Z=3.18 HFD=1.68±0.08 vs. 1.46±0.07	p=0.0015; Z=3.18 HFD=1.55±0.05 vs. 1.46±0.07
Medial segment of precentral gyrus	11		18	p=0.05; Z=1.96 HFD=1.71±0.14 vs. 1.58±0.08	p=0.03; Z=2.13 HFD=1.71±0.14 vs. 1.58±0.06	p=0.004; Z=2.85 HFD=1.71±0.14 vs. 1.42±0.08	p=0.59; Z=0.53 HFD=1.58±0.08 vs. 1.58±0.06	p=0.003; Z=2.93 HFD=1.58±0.08 vs. 1.42±0.08	p=0.003; Z=2.93 HFD=1.58±0.06 vs. 1.42±0.08
Precentral gyrus	10,12		123	p=0.002; Z=3.06 HFD=1.70±0.13 vs. 1.64±0.10	p<0.000001; Z=6.10 HFD=1.70±0.13 vs. 1.58±0.09	p<0.000001; Z=6.74 HFD=1.70±0.13 vs. 1.46±0.09	p<0.000001; Z=5.87 HFD=1.64±0.10 vs. 1.58±0.09	p<0.000001; Z=6.65 HFD=1.64±0.10 vs. 1.46±0.09	p<0.000001; Z=6.74 HFD=1.58±0.09 vs. 1.46±0.09
Superior temporal gyrus	18		79	p=0.005; Z=3.47 HFD=1.56±0.12 vs. 1.56±0.11	p=0.021; Z=1.25 HFD=1.56±0.12 vs. 1.49±0.09	p<0.000001; Z=5.68 HFD=1.56±0.12 vs. 1.39±0.09	p<0.000001; Z=5.63 HFD=1.56±0.11 vs. 1.49±0.09	p<0.000001; Z=5.91 HFD=1.56±0.11 vs. 1.39±0.09	p<0.000001; Z=5.89 HFD=1.49±0.09 vs. 1.39±0.09

brain regions	L	M	N channels	wake - R	wake – N2	wake– N3	R – N2	R – N3	N2 – N3
Middle temporal gyrus	19		126	p=0.27; Z=1.11 HFD=1.56±0.11 vs. 1.54±0.11	p<0.000001; Z=5.06 HFD=1.56±0.11 vs. 1.49±0.08	p<0.000001; Z=7.41 HFD=1.56±0.11 vs. 1.36±0.09	p=0.000001; Z=4.92 HFD=1.54±0.11 vs. 1.49±0.08	p<0.000001; Z=7.42 HFD=1.54±0.11 vs. 1.36±0.09	p<0.000001; Z=7.42 HFD=1.49±0.08 vs. 1.36±0.09
Inferior temporal gyrus	20	20	41	p=0.51; Z=0.66 HFD=1.55±0.11 vs. 1.54±0.06	p=0.0019; Z=3.10 HFD=1.55±0.11 vs. 1.46±0.07	p=0.000005; Z=4.55 HFD=1.55±0.11 vs. 1.35±0.08	p=0.00004; Z=4.12 HFD=1.54±0.06 vs. 1.46±0.07	p=0.000003; Z=4.70 HFD=1.54±0.06 vs. 1.35±0.08	p=0.000003; Z=4.70 HFD=1.46±0.07 vs. 1.35±0.08
Temporal pole and planum polare	21	21	22	p=0.31; Z=1.01 HFD=1.55±0.13 vs. 1.46±0.17	p=0.87; Z=0.17 HFD=1.55±0.13 vs. 1.52±0.10	p=0.03; Z=2.20 HFD=1.55±0.13 vs. 1.38±0.10	p=1.00 Z=0.00 HFD=1.46±0.17 vs. 1.52±0.10	p=0.24; Z=1.18 HFD=1.46±0.17 vs. 1.38±0.10	p=0.018; Z=2.37 HFD=1.52±0.10 vs. 1.38±0.10
Transverse temporal gyrus (Heschls gyrus)			14	p=0.47; Z=0.73 HFD=1.64±0.12 vs. 1.50±0.06	p=0.47; Z=0.73 HFD=1.64±0.12 vs. 1.51±0.06	p=0.14; Z=1.46 HFD=1.64±0.12 vs. 1.45±0.08	p=0.07; Z=1.83 HFD=1.50±0.06 vs. 1.51±0.06	p=0.07; Z=1.83 HFD=1.50±0.06 vs. 1.45±0.08	p=0.07; Z=1.83 HFD=1.51±0.06 vs. 1.45±0.08
Planum temporale			43	p=0.36; Z=0.91 HFD=1.62±0.14 vs. 1.61±0.05	p=0.31; Z=1.02 HFD=1.62±0.14 vs. 1.57±0.06	p=0.0015; Z=3.18 HFD=1.62±0.14 vs. 1.46±0.06	p=0.0007; Z=3.41 HFD=1.61±0.05 vs. 1.57±0.06	p=0.0007; Z=3.41 HFD=1.61±0.05 vs. 1.46±0.06	p=0.0007; Z=3.41 HFD=1.57±0.06 vs. 1.46±0.06
Fusiform and parahippocampal gyri		34,35	45	p=0.005; Z=2.82 HFD=1.50±0.12 vs. 1.54±0.09	p=0.51; Z=0.66 HFD=1.50±0.12 vs. 1.46±0.07	p=0.000009; Z=4.44 HFD=1.50±0.12 vs. 1.32±0.06	p=0.0007; Z=3.41 HFD=1.54±0.09 vs. 1.46±0.07	p=0.000003; Z=4.70 HFD=1.54±0.09 vs. 1.32±0.06	p=0.000003; Z=4.70 HFD=1.46±0.07 vs. 1.32±0.06
Hippocampus		36	36	p=0.31; Z=1.01 HFD=1.59±0.09 vs. 1.61±0.12	p=0.007; Z=2.69 HFD=1.59±0.09 vs. 1.46±0.09	p=0.0015; Z=3.18 HFD=1.59±0.09 vs. 1.40±0.09	p=0.0015; Z=3.18 HFD=1.61±0.12 vs. 1.46±0.09	p=0.0015; Z=3.18 HFD=1.61±0.12 vs. 1.40±0.09	p=0.0015; Z=3.18 HFD=1.46±0.09 vs. 1.40±0.09
Amygdala		37	6	p=0.69; Z=0.40 HFD=1.62±0.08 vs. 1.61±0.13	p=0.04; Z=2.02 HFD=1.62±0.08 vs. 1.50±0.08	p=0.04; Z=2.02 HFD=1.62±0.08 vs. 1.42±0.06	p=0.08; Z=1.75 HFD=1.61±0.13 vs. 1.50±0.08	p=0.04; Z=2.02 HFD=1.61±0.13 vs. 1.42±0.06	p=0.04; Z=2.02 HFD=1.50±0.08 vs. 1.42±0.06

Supplementary Table 1 The results of Wilcoxon tests for each pair of the four vigilance stages (wake, R, N2 and N3) in 38 brain regions listed in the first column. The numbers indicated in the second and the third column correspond to the numbers of the brain areas showed in Figure 1 for lateral (L) and medial (M) sections, respectively. The values of the Z statistic, p-values, and HFD for two compared stages are highlighted in gray for statistically significant differences.
